# A case of endocervical adenocarcinoma detected 16 months after vaginal delivery

**DOI:** 10.1002/ccr3.620

**Published:** 2016-06-30

**Authors:** İpek Ulu, Esra Tuştaş Haberal, Mehmet Serdar Gülşen, Eser Evrim Yoğurtçuoğlu, Gürkan Kıran, Yasemin Çekmez, Gözde Kır

**Affiliations:** ^1^ Department of Obstetrics and GynaecologyÜmraniye Medical and Research HospitalİstanbulTurkey; ^2^ Department of PathologyÜmraniye Medical and Research HospitalİstanbulTurkey

**Keywords:** Endocervical adenocarcinoma, postcoital bleeding, rapid onset, vaginal delivery

## Abstract

The “rapid‐onset” cervical carcinoma is described as the diagnosis of invasive cervical carcinoma within 3 years of a “normal” Pap smear and it is a rare entity. In our case, we aimed to draw attention toward rapid progression of these endocervical adenocarcinomas to macroscopic sizes.

## Introduction

Cervical cancer is the second most common cancer in women worldwide and still remains a leading cause of cancer‐related death for women in developing countries. Although the incidence of invasive cervical cancer has decreased in developed countries due to cytology screening programs 1, the incidence of cervical adenocarcinoma has been increasing especially among younger women 2. In the United States, a relative increase in the incidence of adenocarcinoma of the cervix was detected in the last 20 years 3, which accounts for 24% of all cervical cancers seen in each year 4.

It has been estimated that duration of oral contraceptive use, human papilloma virus type 16 and 18, increasing parity, and younger age at first pregnancy are related to the high rate of cervical adenocarcinomas 3. Earlier neoplastic precursors to cervical adenocarcinoma in situ (AIS) and adenocarcinoma as well as squamous cell carcinoma are not described 5. The “rapid‐onset” cervical carcinoma is described as the diagnosis of invasive cervical carcinoma within 2 or 3 years of a “normal” Pap smear and it is unusual 6. The objective of reporting our case is to draw attention to rapid progression of these endocervical adenocarcinomas to macroscopic sizes.

## Case

**Figure 1:**
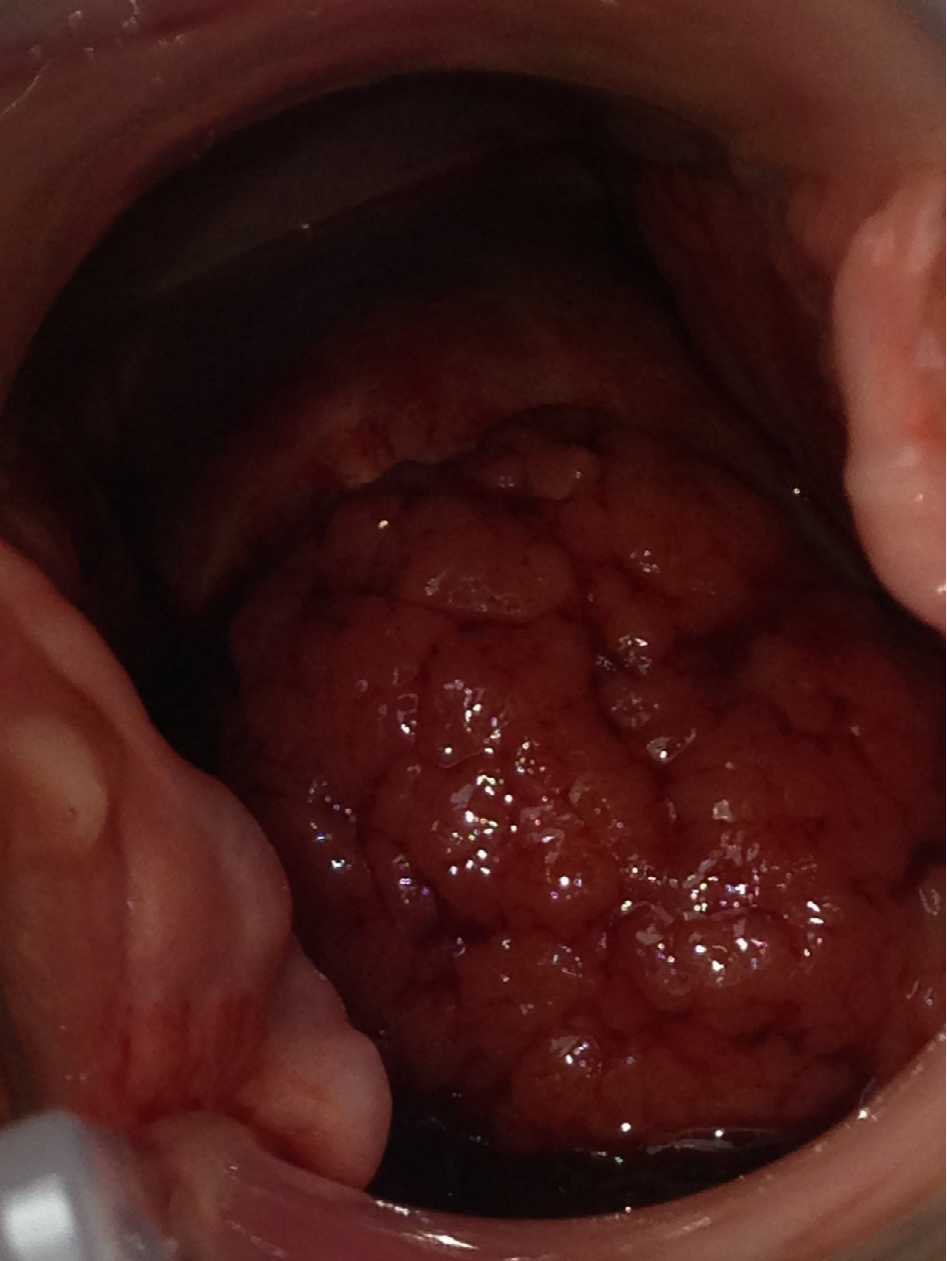
Rapidly progressive cervical adenocarcinoma.

A 37‐year‐old gravida 4, para 4 was referred to our gynecology clinic by her general practitioner 16 months after vaginal delivery, with symptoms of postcoital bleeding for a month. There was no history of itching or offensive smell. She was in the period of lactation without cycle of menstruation. Routine smear test and screening for high‐risk HPV test were both negative 3 years ago. The general practitioner referred the patient to the gynecology clinic after finding an abnormal cervix when he intended to take a smear. The gynecological examination revealed a vegetative mass protruding from cervical canal to vagina, 4 × 3 cm in diameter (Fig. 1).

In the ultrasonographic examination, uterus was retrovert in position, myometrium was homogeneous, the thickness of endometrium was normal, and intrauterine device was seen in the uterine cavity. Magnetic resonance imaging (MRI) showed a suspicious hyperintense 4 × 3 × 1 cm solid mass arising from posterior lip of the cervical canal reaching fornix. No parametrial involvement or significant retroperitoneal lymph node enlargement was detected. Clinical diagnosis of stage 1B2 (International Federation of Gynecology and Obstetrics [FIGO] 2009) was made based on the findings. Punch biopsies of the tumor were taken and sent for histopathological examination.

The diagnosis was moderately differentiated endocervical adenocarcinoma originating from the cervix. Radical hysterectomy with bilateral salpingo‐oophorectomy as well as pelvic and para‐aortic lymph node dissection was performed. The final pathology result was consistent with the previous report which showed moderately differentiated mucinous endocervical adenocarcinoma. The tumor size was 4 × 2.5 × 1 cm. The depth of tumor in the hysterectomy specimen was 1 cm. No parametrial invasion was detected. There was no invasion to uterine corpus or vagina. In total, 30 lymph nodes were extracted. There was no evidence of metastasis to the lymph nodes or lymphovascular invasion. The patient did not receive adjuvant therapy following the hysterectomy.

## Discussion

Cervical cancer is the most common cause of cancer‐related death. The squamous tumors occupy 80% of cervical cancers and the majority of the rest are adenocarcinomas 3. Our case is an example of mucinous endocervical adenocarcinoma which accounts for 70% of all cervical adenocarcinomas 3. Barbu et al. reviewed clinicomorphological data of 16 cases of adenocarcinoma during 2006–2011. The majority of the tumors they reviewed were the mucinous type (62.5%), with the endocervical subtype being the most encountered (43.75%) 3.

The duration of progression is thought to be 5–13 years, during which oncogenic virus infection of the cells in the squamocolumnar junction of the transformation zone leads to the proliferation of atypical glandular cells (AGC) and AIS 5.

The “rapid‐onset” cervical carcinoma is a very rarely seen situation which is defined as the diagnosis of invasive cervical carcinoma following a “normal” Pap smear in 3 years 6. Wain et al. analyzed 237 patients with the histologic diagnosis of invasive carcinoma of the cervix and they detected 51 (21.5%) patients whose smears were reported as “normal” within 2 years of diagnosis 6. They could not find any record of a smear in 15 (29%) patients, although the patients declared the opposite. The slides of six patients had been destroyed and four of these patients had very early adenocarcinomas. They reviewed the slides of the remaining 30 patients and found out that the slides of 16 patients reported as negative, contained cells of in situ or invasive carcinoma. They could not find any cellular material on the slides of four cases. They found atypical cells requesting for further material on the slides of another four cases and detected that these patients were not followed up. Six patients had previously been treated for preinvasive lesions over 3 years. So they stated that they found no case of invasive carcinoma in a patient with a confirmed adequate negative smear within 2 years of diagnosis. They determined that only six (2.5%) patients could have had “rapid‐onset” cancers. They could not confirm it because they could not review these slides. They concluded that the “rapid‐onset” cervical carcinoma was rare in their study 6.

The risk factors of rapid‐onset cervical cancer are similar to those for normal onset cervical cancer 7. Hildesheim et al., in their study of 483 women with the diagnosis of invasive cervical cancer, detected that rapid‐onset cases tended to be younger than normal onset ones 7. They found out that rapid‐onset cases were more likely to be white and diagnosed with adenocarcinomas and especially in early stages. They detected human papillomavirus in 75.2% of cases tested and they suggested that the patients who were positive for human papillomavirus type 18 had a relative risk of 1.6 for rapid‐onset disease. Besides, they pointed that rapid‐onset cancer was not significantly related with any of the risk factors like the oral contraceptive use, cigarette smoking, number of pregnancies, and a maternal history of cervical cancer 7.

Schwartz et al. in their review reported isolated cases diagnosed as invasive cervical cancer within 1 year of a normal Pap smear 8. Second, they reported a 10‐year review of the Yale New Haven Hospital experience that 40 of 555 women had rapidly progressive cervical cancer. In women under 40, 35 (87.5%) cases were detected like our patient who was at the age of 37. They pointed out that these 40 patients diagnosed were due to the persistent symptoms despite a recent normal smear. The final study group in their review included 481 patients who developed cervical cancer in a 5‐year period in Connecticut. Of them, 118 (24.5%) were diagnosed within 3 years of their last true‐negative Pap smear. Adenocarcinomas occurred in 38 (32.2%) patients. They suggested that rapid‐onset cervical carcinoma might be a manifestation of endocervical carcinomas that had been inadequately screened 8.

There are difficulties in the early detection of endocervical adenocarcinoma. Kalir et al. searched whether Pap smears were less effective for the detection of in situ glandular lesions than the detection of squamous counterparts 9. They examined all the pathology materials (reports and slides) including Pap smears and hysterectomy specimens of 53 patients with the diagnosis of endocervical adenocarcinomas at New York University Medical Center. They detected that 10 patients had in situ disease and 7 (70%) of them involving the transformation zone (TZ) were identified by Pap smears. In contrast, of the other three cases that did not involve the TZ and confined to the endocervix, only one was identified by Pap smear. In their study, 43 patients had invasive disease. They revealed that 20 of them involved the TZ, and 23 involved the endocervix but spared the TZ. They showed that 11 (55%) of the cases involving the TZ were identified by Pap smears, whereas 11 (47.8%) of the cases sparing the TZ were diagnosed by Pap smears. They detected that 6 (26%) of the patients with the invasive cancer that TZ was spared had a documented history of negative Pap smears within 3 years of diagnosis. On the other hand, only one of the 20 patients with TZ involvement had a history of negative Pap smears. They concluded that a significantly higher proportion were not detectable by Pap smear if the TZ was spared 9. In our case, the TZ and endocervical cells were involved for screening in Pap smear of our patient.

We aimed to present our case due to the fact that it was a “rapid‐onset” cervical carcinoma. The patient was pregnant 16 months before the diagnosis and no macroscopic tumor was detected during delivery. Routine smear test and screening for high‐risk HPV test of our patient were both negative 3 years prior.

We determined that the “rapid‐onset” cervical carcinoma is a rare entity and most of them are adenocarcinomas. Compared to squamous lesions, Pap smears are less useful for the detection of glandular lesions.

## Conflict of Interest

None declared.
